# Cognitive Factors Related to Drug Abuse Among a Sample of Iranian Male Medical College Students

**DOI:** 10.5539/gjhs.v7n5p143

**Published:** 2015-02-24

**Authors:** Farzad Jalilian, Mari Ataee, Behzad Karami Matin, Mohammad Ahmadpanah, Touraj Ahmadi Jouybari, Ahmad Ali Eslami, Mohammad Mahboubi, Mehdi Mirzaei Alavijeh

**Affiliations:** 1 Substance Abuse Prevention Research Center, Kermanshah University of Medical Sciences, Kermanshah, Iran; 2 Research Center for Behavioral Disorders and Substances Abuse, Hamadan University of Medical Sciences, Hamadan, Iran; 3 Department of Health Education and Promotion, School of Health, Isfahan University of Medical Sciences, Isfahan, Iran; 4 Abadan School of Medical Sciences, Abadan, Iran; 5 Kermanshah University of Medical Sciences, Kermanshah, Iran; 6 Social Determinants of Health Research Center, Yasuj University of Medical Sciences, Yasuj, Iran

**Keywords:** drug abuse, college, student, cognitive factors, Iran

## Abstract

**Backgrounds:**

Drug abuse is one of the most serious social problems in many countries. College students, particularly at their first year of education, are considered as one of the at risk groups for drug abuse. The present study aimed to determine cognitive factors related to drug abuse among a sample of Iranian male medical college students based on the social cognitive theory (SCT).

**Method:**

This cross-sectional study was carried out on 425 Iranian male medical college students who were randomly selected to participate voluntarily in the study. The participants filled out a self-administered questionnaire. Data were analyzed by the SPSS software (ver. 21.0) using bivariate correlations, logistic and linear regression at 95% significant level.

**Results:**

Attitude, outcome expectation, outcome expectancies, subjective norms, and self-control were cognitive factors that accounted for 49% of the variation in the outcome measure of the intention to abuse drugs. Logistic regression showed that attitude (OR=1.062), outcome expectancies (OR=1.115), and subjective norms (OR=1.269) were the most influential predictors for drug abuse.

**Conclusions:**

The findings suggest that designing and implementation of educational programs may be useful to increase negative attitude, outcome expectancies, and subjective norms towards drug abuse for college students in order to prevent drug abuse.

## 1. Introduction

Drug abuse is considered as one of the main social problems worldwide. It ruins lots of lives and huge national capitals are spent to deal with its detrimental consequences on societies. Plus social problems, drug abuse can lead to medical as well as psychological conditions (Scholte et al., 2008). In addition, According to the statistics by the World Health Organization (WHO) reports, drug abuse is increasing among teens and young adults; Iran is no exception; recent studies show that 500 new young Iranian adults start to abuse drugs use a drug every day and addicted population grows twice in each 10 years (Khayatipur et al., 2011). Among young adults, collage students at their first year of education are considered particularly as an in danger group for drug abuse. This is attributed to some factors such as entering a new phase of life, leaving their family, peer tension, etc. (Abbasighahremanloo et al., 2014; Barati et al., 2012; Rezakhani Moghadam et al., 2013). Furthermore, studies have noted that training is the most effective method to prevent drug abuse (Tavousi et al., 2013). On the other hand, designing and editing effective prevention plans require deep understanding of causes of the phenomenon and related factors (Springer et al., 2004). The most efficient training programs stem from theoretical approaches based on behavior change patterns (Tavousi et al., 2013). Among these, a more common pattern and theory to analyze behavior is the social cognitive theory (SCT). Bandura chose to use some concepts of cognitive psychology to combine it with visual learning principles and introduced SCT. Like other behavior psychology theories, SCT is made of several constructs, and this multiplicity makes researchers to choose certain constructs based on the studied behavior (Bandura, 2001).

Considering drug abuse, several studies pointed out to the roles of the constructs of this theory; for example, Gue et al, showed that having positive believes on alcoholic drinks among 10 and 16 year-olds increases the danger of alcoholic drinks abuse at a later time at 21 years of age to 1.2 and 1.7, respectively (Guo et al., 2001). Tucker et al. suggested that having positive attitude to smoking (e.g.: relaxation) increased smoking risk by 1.7 later at 23 years of age (Tucker et al., 2003). Read et al. study on college students attending their first year of education (an average age of 18 years) showed that peer norms had a positive effect on drinking alcoholic drinks among male students (Read et al., 2002). Weintraub Austin and Chen presented similar results about the role of subjective norms in drug abuse (Weintraub Austin, & Chen, 2003). Many studies, also, mentioned the importance of self-control in risky behaviors; for example, Jackson introduced low self-control as an important factor on drug abuse among the youth (Jackson, 2000). In addition, Sussman et al. showed meaningful relation between smoking, using alcoholic drinks and marijuana with low self-control (Sussman et al., 2001). Furthermore, Adalbjarnardottir and Rafnsson in a long-term study suggested that the lower the self-control level, the higher anti-social behaviors among students would be. They also reported higher risk of drug and alcohol abuse among such students (Adalbjarnardottir & Rafnsson, 2002).

Substance abuse, as a non-adaptive pattern of using substances, results in frequent occupational, social, and legal problems (Ahmadpanah et al., 2013). In this regards, drug abuse among college students can be followed several complications, such as reduce academic achievement, educational problems, psychological and physical illnesses, and high risk behavior among students (Barati et al., 2012).

Iran is one of many countries in which the prevalence of the substance abuse has been increasing, especially among the adolescents, young and college students (Ahmadi et al., 2001); furthermore, some study in Iran indicated high rate of substance abuse among medical college students (Homa et al., 2009). Recent studies of the Iranian college students reported high prevalence of the drug abuse, for example 33% (Rezakhani Moghadam et al., 2013), 10% (Serajzadeh, 2007); additionally, Mohammad Khani reported that 18.8 percent of students mentioned experience of substance abuse for at least once (Rezakhani Moghadam et al., 2013; Serajzadeh, 2007; Mohammadkhani, 2012). In other hand, several studies have shown that male students are in higher risk of experiencing and abusing drugs than female students (Chassin et al., 2002; Hicks et al., 2007; Hussong & Chassin, 2004; King & Chassin, 2007; Steinhausen et al., 2008).

Therefore, the main objective of this study was to determine cognitive factors related to drug abuse among a sample of Iranian male medical college students based on the SCT.

## 2. Materials and Methods

### 2.1 Participants and Procedure

This cross-sectional study was conducted on a sample of male students aged 18 to 22 years old with a mean (SD) age of 19.91 (1.19) years in two medical universities in Iran during 2014. The sample size was calculated at 95% significant level according to the results of the previous study (Rezakhani Moghadam et al., 2013) and considering 20% attrition rate, a sample of 425 was estimated. Of the population of 425, 355 of them signed the consent form and voluntarily agreed to participate in the study. Respond rate the response rate was 84%. The study protocol was approved by the institutional review board of the Isfahan University of Medical Sciences; furthermore our research project was approval by the committee of ethics of the Isfahan University of Medical Sciences, the center of Iran.

### 2.2 Measures

Prior to conducting the main project, a pilot study was conducted to assess the utility of the instrument. The pilot study participants were 18 male medical college students, similar to those who participated in the main study. The pilot study was conducted to obtain feedback about the clarity, length, comprehensiveness, and completion time of the various instruments as well as collecting data to estimate the internal consistency of the measures.

### 2.3 Demographics Scale

Background data gathered included age, marital status (single or married), faculty (medical, dentist, pharmacy, health and nutrition, paramedical, nursing), level of parents’ education (primary school, secondary school, high school diploma, and academic education), having friends who had history of drug use (yes, no), having family who had history of drug use (yes, no), parents’ divorce (yes, no), living place (with parents, dormitory, others), and history of persuasion due to drug use.

### 2.4 Drug Use

To assess whether or not the respondents had current drug use, we used their responses to one question: “have you abused drug during the past three months?” The response was “Yes” or “No”.

### 2.5 Predictive Variables (Social Cognitive Theory)

Predictive factors for drug use included some variables of the SCT. This section included 47 items which were composed under six major constructs including (1) attitude towards drug use, (2) outcome expectation towards drug use, (3) outcome expectancies towards drug use, (4) subjective norms toward drug use, (5) behavioral intention toward drug use, and (6) self-control.

Attitude, outcome expectation, outcome expectancies, subjective norms, and behavioral intention towards drug use were designed based on a standard questionnaire related to drug use (McMillan & Conner, 2003; Yeramaneni, 2010; Allahverdipour et al., 2012). Nine items were designed to measure attitude (e.g., drug use for me is: unpleasant-pleasant). Seven items were designed to measure outcome expectation (e.g., if I use drug, I will keep my mind off problems). Seven items were designed to measure outcome expectancies (e.g., how important is it to you that you can keep your mind off problems when you drug use?). Seven items were designed to measure subjective norms (e.g., if I use drug, my best friends will confirm it). Four items were designed to evaluate behavioral intention (e.g., I intend to use drug during university time).

In order to facilitate the participants’ responses to the items, outcome expectation, outcome expectancies, subjective norms, and behavioral intention towards drug use were standardized to a 5-point Likert scale, ranging from 1 (strongly disagree) to 5 (strongly agree). Attitude item was standardized to a 7-point Likert scale, ranging from 1 to 7.

Self-control was measured by the Tangney brief self-control scale (Tangney et al, 2004), and included 13 items, for example: “I am good at resisting temptation”. Using the 1 (not at all) to 5 (very much) scale.

The reliability coefficients for the abovementioned constructs were as follows: attitude (α=0.93), outcome expectation (α=0.90), outcome expectancies (α=0.89), subjective norms (α= .74), behavioral intention (α=0.77), and self-control (α=0.75).

### 2.6 Statistical Analysis

The data were analyzed by the SPSS software for windows (ver. 21.0) using correlation as well as linear and logistic regression at 95% significant level.

## 3. Results

Mean age of the respondents was 19.9 years (range, 18-22 years). The initiation age for drug use was 12. Almost, 4.8 % (17/355) participants were married and 95.2 % (338/355) were single. 3.4 % (12/355) also reported that their parents were divorced. About 44.5% of the respondents (158/355) were freshman students and 55.5% (197/355) were sophomore students. About 3.9% of the respondents (14/355) had history of drug use during the past three months. Furthermore, 12.7% (45/355) stated that they had received suggestions from others to use drugs. Nearly 23.7% (84/355) and 14.9% (53/355) of the respondents reported that their friends and family were having history of drug use, respectively.

Table 1 shows mean (±SD) and bivariate correlations between the behavioral intention to drug use, attitude, outcome expectation, outcome expectancies, subjective norms, and self-control which were all statistically significant at 0.01.

**Table 1:** Predictor variables of drug abuse based on bivariate correlation analysis

	Mean (SD)	X^1^	X^2^	X^3^	X^4^	X^5^
X^1^. Attitude	17.73 (12.61)	1				
X^2^. Outcome expectation	13.21 (6.71)	0.582	1			
X^3^. Outcome expectancies	14.61 (8.42)	0.539	0.555	1		
X^4^. Subjective norms	12.62 (5.27)	0.441	0.353	0.331	1	
X^5^. Self-control	42.34 (8.52)	-0.310	-0.316	-.0222	-.0427	1
X^6^. Behavioral intention	6.52 (3.79)	0.535	0.464	0.404	0.623	-.0394

A hierarchical multiple regression analysis was performed to explain the variation in behavioral intention to drug use. As shown in Table 2, collectively, the SCT variables accounted for 49% of the variation in behavioral intention to abuse drugs among the participant.

**Table 2:** Predictors of the perceptual variables in drug use behavioral intention

Variable	*B*	SE *B*	Beta	*T*	*P*-value
***Step 1***			
Attitude	0.064	0.015	0.213	4.157	0.000
Outcome expectation	0.073	0.029	0.129	2.555	0.011
Outcome expectancies	0.025	0.022	0.056	1.158	0.248
Subjective norms	0.306	0.033	0.425	9.398	0.000
Self-control	-0.042	0.019	-0.094	-2.194	0.029
***Step 2***			
Attitude	0.069	0.015	0.230	4.678	0.000
Outcome expectation	0.084	0.027	0.149	3.136	0.002
Subjective norms	0.309	0.032	0.429	9.526	0.000
Self-control	-0.041	0.019	-0.093	-2.170	0.031
SE=Standard Error Final model: Step 2, Adjusted *R* squared=0.49 and *P*<0.001					

Finally, a logistic regression (backward stepwise method) building procedure was conducted and finally on 3^rd^ step, the procedure stopped and the best model was selected. Among the constructs, attitude, outcome expectancies and subjective norms were the most influential predictive factors for drug use (Table 3).

**Table 3:** Logistic regression analysis for variables related to drug use

Variables	B	S.E.	Wald	Odds Ratio	95% Confidence Intervals		P-_value_
**Step 1**					**Lower**	**Upper**	
Attitude	0.049	0.029	2.823	1.050	0.992	1.111	0.093
Outcome expectation	0.081	0.055	2.160	1.085	0.973	1.209	0.142
Outcome expectancies	0.108	0.060	3.266	1.114	0.991	1.253	0.071
Subjective norms	0.214	0.081	6.945	1.239	1.056	1.453	0.008
Self-control	-0.050	0.053	0.895	0.951	0.857	1.055	0.344
**Step 2**				
Attitude	0.045	0.029	2.467	1.046	0.989	1.106	0.116
Outcome expectation	0.076	0.054	1.969	1.076	0.970	1.201	0.161
Outcome expectancies	0.112	0.060	3.407	1.118	0.993	1.259	0.065
Subjective norms	0.236	0.079	9.026	1.266	1.086	1.477	0.003
**Step 3**				
Attitude	0.060	0.028	4.745	1.062	1.006	1.121	0.029
Outcome expectancies	0.109	0.056	3.754	1.115	0.999	1.245	0.053
Subjective norms	0.238	0.077	9.474	1.269	1.090	1.476	0.002

## 4. Discussion

The results of the study indicated that the positive attitude towards drug abuse, outcome expectancies of drug abuse, and subjective norm encouraging to drug abuse were the three main cognitive factors which were associated with drug abuse among Iranian college students attending medical universities.

Attitude is defined as a person’s beliefs about the results of a behavior and his/her evaluation of them (Ajzen, 1991); therefore, different studies investigated the attitude role in drug abuse (Umeh & Patel, 2004; Ghanizadeh, 2008; Jalilian et al, 2013). The obtained results showed that items such as forgetting problems, improving mental powers and increasing self-esteem received highest average scores among other attitude construct questions. This suggests that drug users had better attitude to positive consequences than negative ones (for example, mental problems). Therefore, it seems that training courses should focus on improving negative attitude on using drug and its negative consequences among students.

Outcome expectancies in the SCT is defined as the values that person places on a given outcomes incentives, and outcomes of change that have functional meaning (Bandura, 2001). Results of the present study showed that items such as being happy, feeling fresh and getting mind away from problems while using drug received highest average score among other constructs of outcome expectancies in using a drug. Callas et al. used the SCT to study factors related to consume alcoholic drinks among high-school students in Vermont, the US and suggested that negative expectancies about alcohol were effective factors on consuming alcohol among teens (Callas et al., 2004). Also, Simon-Morton et al. presented similar results in this field (Simons-Morton et al., 1999). In addition, Lam et al. reported that alcohol consumption was significantly associated with increased positive smoking outcome expectancies (Lam et al., 2014). It is needed to focus on psychic factors, which are interfaces and predicators of behaviors, in comprehensive, sanitary instructive programs (Allahverdipour et al., 2012). In other hand, our findings showed outcome expectation was predict drug abuse behavioral intention, but outcome expectancies was a strong factor for prediction drug use behavior. In this regard Bandura noted that outcome expectancies, occasionally mentioned to as ‘if …then’ statements, are the perceived behavioral and affective consequences of engaging in particular behaviors (Bandura, 2002). Our result confirmed previous research, that positive drug outcome expectancies, was a direct and important predictor of drug use (Alfonso & Dunn, 2007; Connor et al., 2014; Connor et al., 2011). It seem, high positive outcome expectancies (e.g. ‘can participate more at parties when drug use, can overcome shyness, can keep mind off problems) were associated with drug use behavior, but outcome expectation were predictor drug abuse behavioral intention.

Subjective norms are agreed and genesis criteria which regulate people behaviors. Subjective norms are essential factors in human group construction as they represent motivation and route, organize social interactions and make others’ responses predictable and meaningful. Subjective norms introduce correct action methods and show people the things they should avoid. Subjective norms are considered as predictive factors in behavior achievement intention. This construct can be affected by social pressure, or normative beliefs, where its severity depends on people motivation to comply other’s expectations (Ajzen, 1991). Many studies mentioned the effects of subjective norms on forming behaviors related to drug abuse (Jalilian et al, 2014; Leitner et al., 1993; Bashirian et al., 2012; Litchfield & White, 2006). In this regard, Leitner et al. found that 90 percent of participants introduced peer pursers as the main reason to start using drugs (Leitner et al., 1993). Knowing pursers from social and cultural environment of aim group and protective values, positive attitudes and sanitary behaviors and arguing with teens in plans related to them are some of the benefits to use peer group in trainings (Noori Sistani et al., 2010). Efficiency of pursers is the basis of this theory where sensitive information is shared easier among pursers (DiClemente et al., 2001; Karofsky et al., 2001). Trained purser could share information effectively by getting pursers involved and could be more efficient to other pursers by improving; furthermore, a related study showed that only one out of six teens gets information from those others than their pursers (Akbarzadeh et al., 2008). Along with this, Jalilian et al. made use of peer group training approach to prevent anabolic steroid usage and results showed that finishing training program, there was a meaningful decrease in behavioral intention to use anabolic steroids among intervention athletes (Jalilian et al., 2011). Considering the important role of subjective norms in drug abuse and, also, efficiency of using purser training approaches, it is suggested to plan and administer training courses to prevent drug abuse among teens and youth, especially through making use of purser training approach. The present study, also, showed that items such as persuasive environment to use drug and accepting friend’s opinions to avoid drug received higher average scores among other subjective norm items, which represented its importance in planning training programs.

## 5. Conclusion

There are multiple factors to explain or predict the drug abuse among youth people. The present study moderately confirmed the applicability of the cognitive factors to explain drug abuse among medical college students in Iran. Our finding could be useful for guiding practitioners and implementers to design and implement effective preventative programs to protect the youth from drug abuse.
